# Snake Venoms in Drug Discovery: Valuable Therapeutic Tools for Life Saving

**DOI:** 10.3390/toxins11100564

**Published:** 2019-09-25

**Authors:** Tarek Mohamed Abd El-Aziz, Antonio Garcia Soares, James D. Stockand

**Affiliations:** 1 Department of Cellular and Integrative Physiology, University of Texas Health Science Center at San Antonio, San Antonio, TX 78229-3900, USAstockand@uthscsa.edu (J.D.S.); 2 Zoology Department, Faculty of Science, Minia University, El-Minia 61519, Egypt; 3 Department of Pharmacology, Institute of Biomedical Sciences, University of Sao Paulo, Sao Paulo 05508-900, Brazil

**Keywords:** Snake venoms, toxins, pharmacology, therapeutic applications, drug discovery

## Abstract

Animal venoms are used as defense mechanisms or to immobilize and digest prey. In fact, venoms are complex mixtures of enzymatic and non-enzymatic components with specific pathophysiological functions. Peptide toxins isolated from animal venoms target mainly ion channels, membrane receptors and components of the hemostatic system with high selectivity and affinity. The present review shows an up-to-date survey on the pharmacology of snake-venom bioactive components and evaluates their therapeutic perspectives against a wide range of pathophysiological conditions. Snake venoms have also been used as medical tools for thousands of years especially in tradition Chinese medicine. Consequently, snake venoms can be considered as mini-drug libraries in which each drug is pharmacologically active. However, less than 0.01% of these toxins have been identified and characterized. For instance, Captopril^®^ (Enalapril), Integrilin^®^ (Eptifibatide) and Aggrastat^®^ (Tirofiban) are drugs based on snake venoms, which have been approved by the FDA. In addition to these approved drugs, many other snake venom components are now involved in preclinical or clinical trials for a variety of therapeutic applications. These examples show that snake venoms can be a valuable source of new principle components in drug discovery.

## 1. Introduction

There are more than 100,000 venomous animal species identified in the world. Each of these species is capable of producing venoms that often contain upwards to 100 different molecules. Animal venoms are used effectively for defense and predation. Animal venoms are not composed of single toxins but cocktails of complex chemical mixtures of pharmacologically active components including proteins, peptides, and enzymes with specific biological activities, as well as some non-protein compounds such as carbohydrates, lipids, metal ions and other, as yet, unidentified substances [1]. However, venom toxicity is generally linked to few toxins within the venom [2]. Bites or stings by certain venomous animals can result in acute envenomation leading to death [3]. According to the World Health Organization (WHO) recommendation, the most effective treatment for envenomation is the use of an antivenom serum [4]. To enhance the efficacy of such managements, a better information about venom composition is required.

Despite their name, most toxins are not toxic, and many have beneficial therapeutic applications. Forty percent of marketed drugs target G protein coupled receptors, and ion channels are the second most important class of drugable receptors. Since cell surface expressed receptors are major classes of pharmacological intervention, there is little surprise that many toxins became marketed drugs for various pathological conditions. Since these compounds are active, it is fair to consider animal venoms as natural mini bio-libraries that may have a tremendous potential for the identification of new drugs [5]. The major difference between these natural libraries and libraries of synthetic compounds often employed for pharmaceutical research is that the former contains compounds known to be biologically active. Investigation on venom composition converges towards the general concept that venoms are composed of about 100 to 500 pharmacologically active compounds. Considering these numbers, there are about 10 to 50 million natural compounds that can be used for drug discovery. Nevertheless, less than 0.01% of these compounds have been identified and characterized and a large proportion of toxins act on unknown receptors. Moreover, most known toxins have been described only incompletely. The reasons for this are well understood: Difficulties in obtaining reliable sources of venoms, the use of inadequate screening tests, difficulties in purifying and characterizing in detail a given toxin, the limited number of academic or industrial groups working on this type of research. However, the numbers of such groups are steadily increasing, reflecting the dynamics and importance of this sector. In addition, the use of animal venom components as a natural product resource of materials for biotechnological applications has received much attention, recently, from pharmaceutical industries and experts in the fields of applied research [6,7].

**Figure 1:**
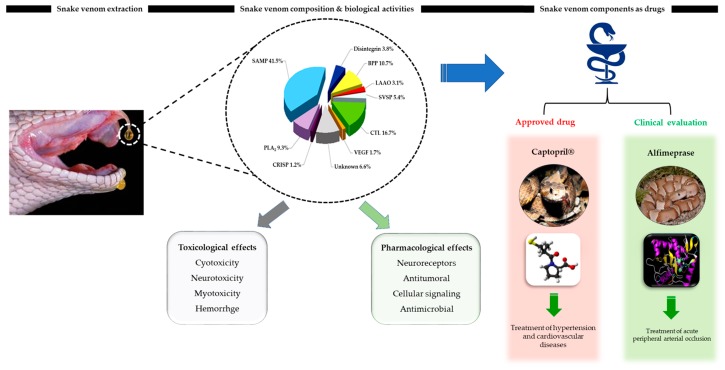
A schematic representation of snake venoms, aiming to assess the significance of snake venoms as a resource of novel drugs. Snake Venom Metalloproteinase (SVMP), Phospholipase A2 (PLA2), Cysteine-rich secretory protein (CRISP), Vascular Endothelial Growth Factor (VEGF), C-type lectin-like toxin (CTL), Serine proteinase (SVSP), L-amino acid oxidase (LAAO), Bradykinin Potentiating Peptide (BPP).

Animal venom studies initially began with the enthusiasm of understanding animal envenomation and associated medical treatments [8]. Subsequently, additional reasons that make animal venoms attractive to researchers around the world are the richness, specialization, and efficiency of their components: most of these components are peptides that affect with high selectivity and affinity a large number of targets such as membrane receptors, ion channels, enzymes or various hemostatic pathways [9]. From the early 17th century, the Italian naturalist Felice Fontana illustrated the influence of snake venoms on blood coagulation [10]. Currently, animal venom components are being used as valuable and powerful pharmacologically research tools (Figure 1). Venom derived-drugs have been produced by the pharmaceutical industry as Captopril, Aggrastat, and Eptifibatide, all designed based on snake venom components [11]. Many additional animal venom components are also currently in different clinical stages as therapeutic drugs. Finally, there are many venomous organisms in the animal kingdom, such as snakes, scorpions, spiders, insects, amphibian and cone snails, and their venoms may be useful in biotechnological or pharmacological applications.

## 2. Classification and Distribution of Venomous Snakes

**Figure 2:**
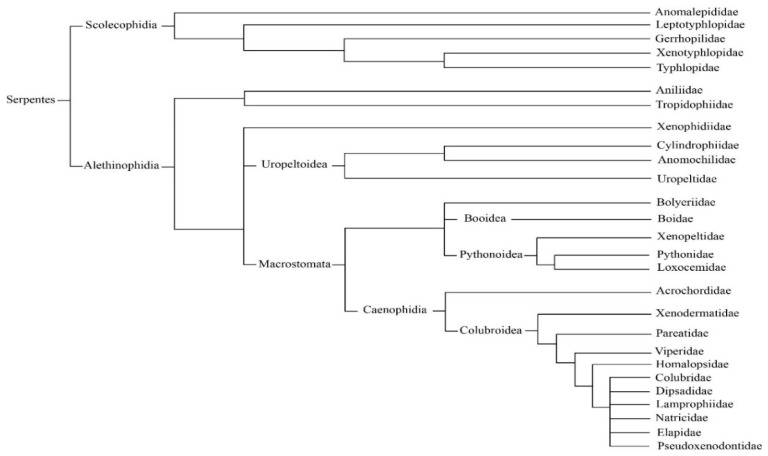
Phylogeny of snake species.

There is competition for resources between the different living organisms on our planet and the ecosystem supporting life of venomous organisms and always survival of the fittest [12]. Venomous animals are a subject of fear and fascination for many people in the world. Venomous animals distribute in several regions around the world especially in tropic and subtropics regions. It is now accepted that there are more than 100,000 venomous animal species throughout the world. Only a very small fraction of these animals have been studied with regard to their venom composition [13]. Most of the venomous animal species are found in multiple phyla such as Chordata (reptiles, fishes, and amphibians), Arthropod (arachnids and insects), Mollusca (cone snails), Echinodermata (starfishes and sea urchins), and Cnidarian (sea anemones, jellyfish and corals) [14]. Furthermore, venomous animals are defined as those that inject their venoms into other living organisms using different apparatus such as spurs, stingers, spines, or fangs [15]. Venoms are secreted and delivered through well-developed venom exocrine glands and coupled to a delivery system, which have different vital functions for these animals that include capturing, killing and digesting prey, but they can also be considered as a defense mechanism against potential enemies [16]. Snakes are known as a subject of fascination, fear, and folk stories throughout history. The cobra was worshipped in ancient Egypt, and Roman emperors used the form of the cobra to decorate their crowns. All snakes are classified within the suborder Serpentes of the reptilian order Squamata, named for their scaly skin [17]. The two major infraorders Scolecophidia and Alethinophidia include around 3600 species located in approximately 27 families (Figure 2). The superfamily Colubroidea (>2500 species) are known today as advanced snakes and includes the majority of snake species [18]. Colubroidea includes several families including Viperidae, which contains around 331 species of vipers and pit viper snakes. It is the most prevalent family of venomous snakes with distribution throughout Europe, Africa, Asia, and the Americas, but absent in Australasia. Elapidae consists of 358 species of kraits, corals, mambas, and cobra snakes. Elapidae snakes are distribute worldwide with representatives in Africa, the Americas, Asia, and Australasia. However, they are most abundant in tropical and subtropical regions. Colubridae contains about 847 species of rear-fanged and harmless colubrid. It is widely distributed in all parts of the world. Lamprophiidae includes 309 species of Stiletto snakes and mole vipers. These are found in Africa, Asia, Europe and the Americas (http://www.reptile-database.org).

## 3. Composition of Snake Venoms

**Figure 3:**
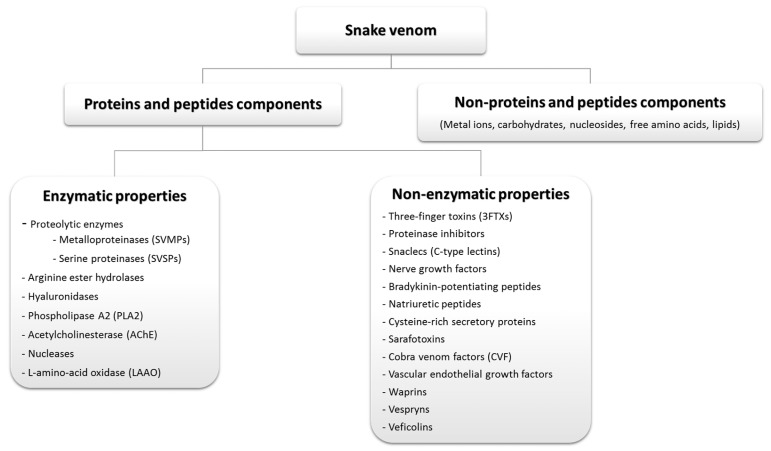
Snake venom composition.

Traditionally, people believe that snakes are dangerous and represent a threat to their lives with little appreciation for the fact that snake venom components can represent beneficial medical tools for the treatment of human diseases. Venomous snakes are well known as rich sources of toxins among other venomous species and their venoms are the most highly developed and extremely complex of all natural venoms [19]. Generally, snake venoms are cocktail secretions produced by a pair of specialized exocrine venom glands connected to the fangs by ducts. Snake venoms are not composed of a single component but instead are complex mixtures of toxic and biologically active proteins and peptides. While some reports demonstrated that around 100 components are present in single venom, it is not known exactly how many proteins and peptides are present in snake venom, but it probably is upwards to about 90–95% of the dry weight of the venom. Some of these proteins exhibit enzymatic activities, whereas several others are non-enzymatic proteins and peptides (Figure 3). Other components in the snake venom are nucleosides, metallic cations, carbohydrates and very low levels of free amino acids and lipids with less biological activity [20]. Sodium is the most abundant cation in snake venom, but its role is unknown. Zinc is needed for activation of anticholinesterase (acetylcholinesterase inhibitor) and calcium is required for phospholipase activity [21]. The presence of cadmium was found to inhibit biological processes in specific enzyme activities. Variation in snake venom composition is found between species, subspecies or even in the same snake specimen [22]. Snake venom variation is associated with many factors including phylogeny [23], geographic distribution [24], age [25], sex [26], and diet [27].

### 3.1. Enzymatic Proteins from Snake Venoms

Snake venoms are cocktails, which exhibit enzymatic activities. Most commonly, snake venoms possess enzymes such as proteolytic enzymes, arginine ester hydrolase, thrombin-like enzymes, hyaluronidase, phospholipase A_2_, acetylcholinesterase, nucleases (RNase, DNase, and Phosphodiesterase), and L-amino-acid oxidase. Snake venoms are known as some of the richest sources of these enzymes [28].

#### 3.1.1. Proteolytic Enzymes

Most snake venoms contain proteolytic enzymes. These enzymes catalyze the digestion of tissue proteins and peptides into amino acids. Proteolytic enzymes can be classified into two major groups: metalloproteases and serine proteases, which affect the hemostatic system through different mechanisms [29]. These proteolytic activities are reported in the snake venoms of Elapidae and Viperidae families. The Australian red bellied black snakes *Pseudechis porphyriacus* and *Pseudechis australis* showed high levels of protein digestion action indicating that there is at least one active protease in their venom [30]. Snake venom metalloproteinases (SVMPs) are one of the main proteolytic enzymes contributing to crotalid and viperid snake venom toxicities. Their catalytic activity is dependent on the presence of zinc ions. SVMPs represent one of the most abundant components of the Crotalidae and Viperidae venoms with varying percentage from 11% to 65% of the total proteins in these venoms [31]. Other studies showed that SVMPs can be detected in lower amounts in the snake venoms of the Elapidae family [32]. Such observations are consistent with these proteases playing critical roles in envenomation-related pathogenesis to include bleeding, intravascular clotting, edema, inflammation and necrosis. SVMPs are mainly known for their pro-hemorrhagic activities and may interfere with the hemostatic system. However, there are a number of other activities related to the presence of SVMPs such as fibrin(ogen)olytic activity, prothrombin activating, platelet aggregation inhibition, and inactivation of blood serine proteinase inhibitors [33,34,35]. Because SVMPs degrade the main components of the capillary basement membrane, they cause the escape of blood content from the intravascular space to surrounding tissues [36]. SVMPs are classified into P-I, P-II, P-III, and P-IV classes depending on their size and domain structure organization [37].

Snake venom serine proteinases (SVSPs) are present mainly in the venoms of Viperidae, Crotalidae, Elapidae, and Colubridae snakes and are more rarely detected in the venoms of Hydrophiidae snakes [38]. SVSPs are well-studied venom enzymes affecting generally the hemostatic system. Individually, they are not considered lethal, but they contribute to the toxic effect when combined with other proteins of the venom [39,40]. Fundamentally, SVSPs can be categorized into different subtypes depending on their mode of action to include thrombin-like enzymes, kallikrein-like, plasminogen activators, platelet aggregation inhibitors, protein-C activators and prothrombin activators [41]. Snake venom thrombin-like enzymes (SVTLEs) possess coagulant activities similar to thrombin but are not inhibited by heparin and they do not activate the blood coagulation factor XIII [29]. They convert fibrinogen to fibrin by the cleavage of the Aα and Bβ chains and in vitro increase clotting. However, in vivo, they have alternative effects to include hemostasis and anticoagulation [42]. To date, a large number of studies have led to the isolation and identification of TLEs mainly from the venom of the subfamilies Viperinae and Crotalinae [43]. Many TLEs have been isolated, such as Ancrod isolated from *Agkistrodon rhodostoma* [44], Crotalase purified from *Crotalus adamanteus* [45], ABUSV-SPase characterized from *Agkistrodon blomhoffii ussurensis* [46], Calobins isolated from *Agkistrodon caliginosus* [47], Elegaxobin purified from *Trimeresurus elegans* [48] and BjussuSP-I from *Bothrops jararacussu* [49]. Kallikrein-like serine proteases cause release of bradykinin from the high molecular weight kininogen and the degradation of angiotensin [50].

#### 3.1.2. Arginine Ester Hydrolases

Arginine ester hydrolases are widely distributed in the venoms of Crotalidae and Viperidae snakes but are not present in the venoms of Elapidae and Hydrophiidae snakes [51]. They cause hydrolysis of the ester or peptide linkage to which an arginine residue contributes the carboxyl group. Arginine ester hydrolase activity is frequently accompanying with the presence of thrombin-like and kinin-releasing enzymes in snake venoms. This activity has been best described in some crotalid snake venoms to include that from *Agldstrodon halys blomhoffii*, *Agkistrodon contortrix iaticinctus*, *Crotalus adamanteus, Trimeresurus flavoviridis, Hypnale hypnale,* and *Bothrops Jararacussu* [52,53].

#### 3.1.3. Hyaluronidases

Hyaluronidases are classes of endoglycosidases that are present in almost all snake venoms. They are considered to be “spreading factors”. They damage the extracellular matrix at the site of a bite leading to severe morbidity [54]. This not only facilitates the distribution of other toxins but also damages the local tissue of the victims [55]. Hyaluronidase activity is found in the venoms of Elapidae, Viperidae, and Crotalidae snakes [56].

#### 3.1.4. Phospholipase A_2_ (PLA_2_)

Phospholipases A_2_ (PLA_2_s) enzymes, which play essential roles in various biological activities, are common to many snake venoms. Phospholipases are a class of enzymes that have the ability to hydrolyze specific substrates like glycerophospholipids. To date, they are recognized to be in four families A1, A2, C, and D. This classification identifies the site of hydrolysis on the substrate [57]. Among these families, PLA_2_s are the best studied due to their wide distribution in nature and PLA_2_s have been classified into more than 15 groups including several subgroups [58]. In the case of snakebites, PLA_2_ enzymes play a significant role in the digestion of the prey in addition to a wide variety of other pharmacological effects by producing changes in the permeability of cell membranes by cleaving the sn-2 bond in glycerophospholipids to release free fatty acids and lysophospholipids [59,60]. PLA_2_s catalyze the Ca^2+^-dependent hydrolysis of the acyl ester at the sn-2 position of glycerophospholipids. PLA_2_s have been identified in almost all snake venoms of Viperidae, Hydrophidae, and Elapidae families [61].

#### 3.1.5. Acetylcholinesterase (AChE)

Acetylcholinesterase is present in all vertebrate organisms, mostly located in the muscles and nervous tissues. AChE plays a central role in cholinergic transmission by rapidly hydrolyzing the neurotransmitter, acetylcholine, into choline and acetic acid [62]. High AChE activity was described in all Elapid snake venoms, excluding those from the mamba species. In particular, the venom of the *Bungarus* genus of Elapidae contains high level of AChE activity since significant quantities can be purified from the crude venom (about 8 mg/g in dried venom) [63]. On the other hand, AChE activity has not been detected in any venom from the Viperidae or Crotalidae families [64].

#### 3.1.6. Nucleases (RNase, DNase, and Phosphodiesterase)

Nucleases (DNase, RNase, and phosphodiesterase) are enzymes present in almost all snake venoms that specifically hydrolyze nucleic acids (DNA and RNA) [65]. Snake venom nucleases are subdivided into endonucleases (DNase, RNase) and exonucleases (phosphodiesterases) [66,67]. To date, only a few studies have been performed on snake venom deoxyribonucleases (DNases). Consequently, little is understood about these proteins. DNases have been isolated from *Bothrops atrox*, *Bothrops schlegelii,* and *Vipera lebetina* venoms that breakdown the DNA structure [65,68,69]. The activity of ribonuclease enzymes was found to be particularly high in the venom of *Naja oxiana* compared to other snake venoms [70]. Similarly, an RNase was isolated from *Naja naja* venom with specificity for polycytidine [71]. Compared to DNase and RNase, snake venom phosphodiesterases (PDEs) have been widely studied [66]. PDEs have been purified and characterized from several species of snakes from Crotalidae and Viperidae families, whereas venoms of the Elapidae and Hydrophidae have low levels of this enzyme [72,73]. Theses enzymes break phosphodiester bonds and catalyze the hydrolysis of DNA, RNA, or any nucleotide chain [74].

#### 3.1.7. L-amino-Acid Oxidase (LAAO)

L-amino-acid oxidases are flavoenzymes that stimulate the stereospecific oxidative deamination of an L-amino acid and act as a substrate for α-keto acid producing ammonia and hydrogen peroxide [75]. The enzyme is common in nature to include being found in a variety of snake venoms. LAAOs occur in several venoms of Viperidae and Elapidae venomous snakes but the richest sources of LAAOs are the Crotalidae snake venoms [76]. Recently, LAAOs have become an interesting source for biomedical research because of their anticoagulant, antimicrobial, platelet aggregation-inducing and inhibiting, apoptotic-inducing, and anti-cancer activities [77,78].

### 3.2. Non-Enzymatic Proteins from Snake Venoms

**Figure 4:**
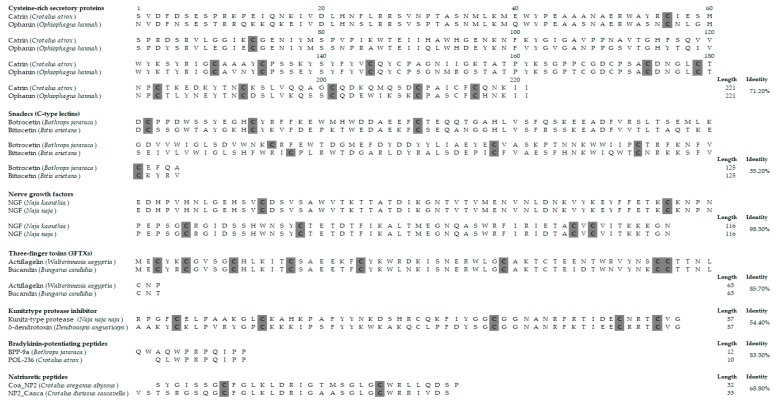
Amino acid sequences of snake venom non-enzymatic proteins and peptides. Sequence alignment of two proteins for each non-enzymatic family [82,86,87,88,89,90,91,92,93,94,95,96]. All cysteine residues are shaded in grey. The peptide lengths and percentages of sequence identities are given on the right.

Studies in the past few decades have revealed that snake venoms are also rich in non-enzymatic proteins. In the opposite of the snake venoms enzymatic proteins, non-enzymatic proteins contribute generally to immobilization of prey [79]. In general, they act on specific membrane receptors, ion channels or plasma proteins, which cause disruption of the physiological processes of the prey by leading to neurotoxic and cardiotoxic effects [80]. They are categorized into many protein families depending on their amino acid sequences and protein folding. Among the well-known non-enzymatic proteins families in snake venoms are (i) cysteine-rich secretory proteins (CRISPs) or helveprins; (ii) snaclecs (C-type lectins and related proteins); (iii) proteinase inhibitors; (iv) nerve growth factors; (v) bradykinin-potentiating peptides; (vi) natriuretic peptides; (vii) three-finger toxins; (viii) sarafotoxins; (ix) cobra venom factors; (x) vascular endothelial growth factors; (xi) vespryns; (xii) waprins; and (xiii) veficolins [81,82,83,84]. Within each family, the non-enzymatic proteins and peptides share remarkable similarities in their primary, secondary, and tertiary structures, but they may differ from each other in their pharmacological effects (Figure 4) [80,85].

## 4. Pathophysiological and Pharmacological Actions of Snake Venoms

The summated biological properties of snake venoms closely follow that of their constituent components. Several snake venom components including PLA_2_, serine proteases, metalloproteinase, lectins, l-amino-acid oxidases, bradykinin potentiating factors, natriuretic factors, and integrin antagonists have valuable natural pharmacological actions that induce neurotoxicity, myotoxicity, cytotoxicity, hemotoxicity, and antimicrobial activity [2,97]. In general, snake venom components interact with a wide variety of mammalian proteins and can disrupt the central and peripheral nervous systems, the blood coagulation cascade, the cardiovascular and neuromuscular systems, and the general homeostasis state.

### 4.1. Neurotoxicity

Neurotoxicity is a well-known feature of snake envenomation. Neurotoxins were first purified from snake venoms approximately 50 years ago. The majority of the neurotoxins (e.g., ion channel blockers and membrane receptor blockers) act on the peripheral nervous system where the skeletal neuromuscular junction is a favorite target. Neurotoxins lead to acute neuromuscular paralysis, a cause of morbidity and mortality [98]. This has been understood to be a mechanism of action for toxins in Elapidae snake venoms to include that form kraits (*Bungarus* species), cobras (*Naja* species), death adders (*Acanthophis* species), taipans (*Oxyuranus* species), coral snakes (*Micrurus* species), and tiger snakes (*Notechis* species) [99,100]. Recently, this has also been discovered in Viperidae snake venoms to include that of rattlesnakes (*Crotalus* species). Neurotoxicity is well known in envenoming by viper snakes to include Russell’s viper (*Daboia russelii*), asp viper (*Vipera aspis*), adder (*Vipera berus*), and nose-horned viper (*Vipera ammodytes*) [101,102]. Traditionally, snake venom neurotoxins are known to produce two types of neuromuscular blockade: presynaptic (β-neurotoxins) and postsynaptic (α-neurotoxins) according to their site of action.

#### 4.1.1. Presynaptic Neurotoxins

Snake venom toxins affecting the release of acetylcholine (Ach) from the presynaptic membrane are known as presynaptic neurotoxins (β-neurotoxins) [103]. Several presynaptic neurotoxins are related to the activity of phospholipase A_2_ (PLA_2_) enzymes in snake venoms. These β-neurotoxins play a critical role in snake envenomation blocking transmission at the neuromuscular junction without affecting the sensitivity of the motor end plate to ACh. They are responsible for high toxicity and respiratory failure [104]. They have been identified in venoms from four major families of venomous snake: Crotalidae, Elapidae, Hydrophiidae, and Viperidae. Presynaptic toxins are well explained by β-bungarotoxin (β-BuTX) of the kraits snake (*Bungarus multicinctus*) which mainly has PLA_2_ enzymatic activity [105,106]. Their structures vary from single-chain polypeptides (notexin, from *Notechis scutatus*) to toxins consisting of multiple subunits. For example, crotoxin (from *Crotalus durissus terrificus*) consists of two subunits, taipoxin (from *Oxyuranus scutellatus*), paradoxin (from *Oxyuranus microlepidotus*), and cannitoxin (from *Oxyuranus scutellatus canni*) consist of three subunits and textilotoxin (from *Pseudonaja textilis*) contains four subunits [107,108,109].

#### 4.1.2. Postsynaptic Neurotoxins

Neurotoxins affecting the postsynaptic membrane are commonly called postsynaptic neurotoxins (α-neurotoxins). α-neurotoxins prevent the opening of ion channels and disrupt neuromuscular transmission by binding with high affinity to postsynaptic nicotinic acetylcholine receptors (nAChRs) at the skeletal neuromuscular junction [110,111]. This leads to death by asphyxiation. To date, more than 100 α-neurotoxins have been identified and their amino acids sequences have been determined but only a few thus far have been characterized pharmacologically [112]. In contrast with β-neurotoxins, most of the α-neurotoxins are isolated from the snake venoms of the Elapidae and Hydrophiidae families (*Naja*, *Bungarus* and other species) [103]. Recently, α-neurotoxins have also been discovered in colubrid snake venoms to include α-colubritoxin (isolated from the Asian rat-snake *Coelognathus radiatus*), rufoxin (identified in the venom of the Rufous break snake *Rhamphiophis oxyrhynchus*), boigatoxin-A, and denmotoxin (characterized from the venom of the Mangrove cat-snake *Boiga dendrophila*) [113,114]. According to their sequence, α-neurotoxins are subdivided into two major groups, short-chain toxins consisting of between 60 and 62 amino acid residues organized in a single chain and cross-linked by four disulphide bridges; and long-chain toxins that have 66–74 amino acid residues and also organized into a single chain but cross-linked by five disulphide bridges [115]. From the perspective of research, α-neurotoxins have been very helpful in understanding the structure and function of AChRs. For example, α-Bungarotoxin played a significant role in understanding the pathology of myasthenia gravis, a disease that causes weakness of skeletal muscles. However, there are no drugs currently used in the clinic based on α-neurotoxins [116].

#### 4.1.3. Dendrotoxins

Dendrotoxins and related peptides are small non-enzymatic venom neurotoxin proteins that were purified from green mamba snake venoms (*Dendroaspis angusticeps*) and the venoms of related mambas (*Dendroaspis* sp.) [117]. Dendrotoxins have a full sequence of 57–60 amino acids cross-linked by three disulfide bonds [118]. Several homologues, including α-, β-, γ-, and δ-dendrotoxins can be present in the same snake venom [119]. Dendrotoxins have been found to be valuable therapeutic agents due to their high potency and selectivity for neuronal potassium channels [120]. Several studies have shown that dendrotoxin from the green mamba blocks distinct potassium channels to include Kv1.1, Kv1.2, and Kv1.6 channels [121,122].

### 4.2. Hemotoxicity

Hemotoxicity is mainly caused by anticoagulant, procoagulant, fibrinolysins, hemorrhage, and hemolysins factors. Snake venoms of Viperidae and Crotalidae families are a rich source of proteins and peptides that interact with the components of the hemostatic system, resulting in hemorrhage that can be witnessed after snakebites [123]. These components (enzymatic and non-enzymatic) can be categorized into coagulant, anticoagulant, and fibrinolytic factors [124,125]. The Italian Fontana, was the first scientist to detect the coagulant properties of viper venom near the end of the 1700s [126]. Several studies indicate that more than 100 snake venom components act on the hemostatic system through different mechanisms [125,127,128].

#### 4.2.1. Pro-Coagulant Activities

Several venoms components act on elements of the coagulant cascade and activate the coagulation system. These venom proteins include different activators of blood coagulation factors such as factor V, IX, X, and prothrombin activators [129]. Not all snake species contain each of these activators. For instance, venom of the Russell’s viper (*Daboia russelli*) contains coagulation activators for factor V and factor X but not IX [130]. Moreover, activators of blood coagulation factor X have been isolated from the venom of several snake species within the Viperidae, Crotalidae families as well as from few elapids. Viperid and crotalid venom activators are generally metalloproteases but elapid venom activators are serine proteases. Activators of factor X have been reported in the venom of *Bothrops atrox* [131], king cobra (*Ophiophagus hannah*) [132], banded krait (*Bungarus fasciatus*) [133], Levantine viper (*Vipera lebetina*) [134], and other species. In the clotting process, factors V, IX, X along with calcium ions and phospholipid, activate prothrombin and induce fibrin clot formation. Therefore, snake venom activators play an significant role in the blood coagulation process and can provide a potential source for the expansion of new therapeutic agents [135].

#### 4.2.2. Anticoagulant Activities

Several studies indicate that snake venom toxins (proteins with molecular weights ranging from 6 to 350 kDa) function as direct or indirect anticoagulants by inhibiting the clotting process [128,136]. Consequently, they are often responsible for bleeding associated with envenomation. The anticoagulant action of snake venom proteins is due to protein C activators, blood coagulation factors IX and X inhibitors, and the inhibition of thrombin or phospholipases [123,127]. Venom anticoagulant proteins can be enzymes, as discussed above, to include PLA_2_s and proteinases, or non-enzymatic proteins. PLA_2_s are classified into different categories: strongly, weakly and non-anticoagulating enzymes according to their anticoagulant properties. PLA_2_s prevent formation of the prothrombinase complex by degrading phospholipids within this complex [137]. Protein C activators (Protac) were isolated and characterized from the venom of southern copperhead (*Agkistrodon contortrix contortrix*) and other *Agkistrodon* species indicating that these venoms have anticoagulant activities [138,139,140]. Factors X and IX binding proteins were identified in the venoms of hundred-pace snake (*Deinagkistrodon acutus*), habu snake (*Trimeresurus flavoviridis*), Stejneger’s bamboo pit viper (*Trimeresurus stejnegeri*), and saw-scaled viper (*Echis carinatus leucogaster)* [141,142,143]. These binding proteins bind specially to the Gla-domain (4-carboxyglutamic acid residues) of factors X and IX preventing these factors from recognizing phosphatidylserine on the plasma membrane [144]. Bothrojaracin a thrombin inhibitor was isolated and characterized from the venom of *Bothrops jararaca* [145]. It is believed that further identification of the mechanisms of action of these anticoagulant toxins will be helpful in designing novel anticoagulant compounds inspired by snake venom toxins.

#### 4.2.3. Fibrinolytic Factors

Several recent studies have attempted to describe the snake venom fibrinolytic enzymes that have been isolated from the venoms of Asian, North and South American crotalid species, including copperheads and rattlesnakes, but also detected in the venoms of cobras within the Elapidae family snakes [146]. Fibrinolytic enzymes can be further classified into *α*- or *β*-chain fibrin(ogen)ases according to their specificities. Most of the venom fibrinolytic enzymes are considered metalloproteinases. The fibrinolytic metalloproteinase isolated from the venom of desert adder (*Vipera lebetina*) directly acts on the fibrin(ogin) *α*-chain more than the *β*-chain [147]. Low molecular weight fibrinolytic enzymes, jararafibrases I, II, III, and IV isolated from the venom of *Bothrops jararaca* exhibited a hemorrhagic activity [148,149].

### 4.3. Cytotoxicity

Cytotoxic snake venoms target specific cellular sites. These cytotoxins can interact with lipoproteins present in the plasma membrane of cells to cause shrinkage [150,151]. Thus, the cytotoxic effects of snake venom components have the potential to degrade/destroy tumor cells. Elapid venoms is possessed significantly cytotoxicity towards both B16F10 melanoma and chondrosarcoma cell lines than that of viperid or crotalid venoms [152]. Elapid venoms disrupted the cell membrane leading to cell death. Currently, cancers are one of the major public health problems around the world and finding new cancer treatments is a major research focus worldwide. Several studies have found that snake venoms possess therapeutic agents that can be used as anticancer agents [153,154]. Therefore, snake venoms could open the doors for novel areas of drug development and research for new cancer treatment [155]. Many excellent publications characterized the use of venom compounds from *Bothrops newweidii*, *Naja naja*, *Naja nigricollis*, *Naja naja atra*, *Bothrops leucurus*, *Laticauda semifasciata Opiophagus hannah*, *Bothrops jararacussu*, *Daboia russelli russelli, Lapemis curtus*, *Bungarus multicinctus, Walterinessia aegyptia, Crotalus durissus terrificus, Agkistrodon acutus, Macrovipera lebentina, Bungarus fasciatus, Lachesis muta muta,* and *Agkistrodon rhodostoma* for the treatment of various conditions to include cancer and inflammation [156,157,158].

### 4.4. Myotoxicity

Local and systemic skeletal muscle degeneration are associated with some snakebites. Myotoxins are found in snake venoms, which have specific actions on the skeletal muscle affecting the integrity of the sarcolemma, resulting in hemorrhage and necrosis [159,160]. Myotoxins have been isolated from elapid and viperid snake venoms and are rich in phospholipases. PLA_2_ are the most important and abundant myotoxins in these snake venoms [159,161]. However, there are other examples of myotoxins such as low molecular mass myotoxins (myotoxin-a, crotamine) isolated from the Prairie rattlesnake (*Crotalus viridis viridis*) and the South American rattlesnake (*Crotalus durissus*) venoms that reportedly bind specifically to sodium channels. Some snake venoms also contain cardiotoxins, including polypeptides of 60–65 amino acids purified from cobra venoms that cause the depolarization and degradation of the plasma membrane of skeletal muscle cells [162,163].

### 4.5. Antimicrobial

Antimicrobial factors are used for the treatment of microbe infections such as viruses, protozoa, bacteria, and fungi (Table 1). Bacteria are the biggest and most wide group of pathogenic microorganisms. Bacterial infections are one of the important causes of death and an vital health problem in need of new sources of antibacterial agents [164]. Many antimicrobial studies show that a large number of snake venom components have antibacterial properties, to include L-amino acid oxidase and PLA_2_ that hydrolyze phospholipids and could possibly act on the bacterial cell surface [165,166,167,168]. Such snake venom PLA_2_ has been purified from the venom of *Bothrops* species (*B. asper*, *B. jararacussu*, *B. pirajai*, and *B. moojeni*), *Bungarus faciatus,* and *Crotalus durissus terrificus* [169,170], whereas such L-amino acid oxidases have been purified from the venom of *B. alternatus*, *B. pirajai, B. asper*, *B. leucurus*, *Crotalus adamanteus,* and *Pseudechis australis* [171,172]. Finally, several studies indicate that venom antibacterial activities are generally dependent on the venom compounds and bacterial types [173].

Few published studies report the ability of using snake venom components as an antiviral agent. Non-cytotoxic, crotoxin and PLA_2_ components were isolated from the venom of *Crotalus durissus terrificus* and their effects against measles, yellow fever and dengue viruses reported [174,175]. Cytotoxins from *Naja nigricollis* venom were shown to possess antiviral activity against the Sendai virus [176]. Moreover, venoms of different snakes, including *Bungarus candidus*, *N. naja, Trimeresurus stejnegeri,* and *N. kaouthia*, have demonstrated anti-HIV activity [177,178,179]. Recently, a study showed the antiviral effects of the crotoxin, phospholipase A2 and crotapotin components isolated from *Crotalus durissus terrificus* venom on hepatitis C virus (HCV) life cycle [180]. In recent decades, the crude venom of *Crotalus durissus cumanensis* has been shown to have antifungal activity since several venom components inhibit the growth of fungi. Antifungal activity may be associated with the cytotoxicity effects of metalloproteinases and phospholipase [181]. The antifungal activity of the rattlesnake venom (*Crotalus durissus terrificus*) appears to be due to crotamine (a small basic polypeptide) [182]. Several snake venoms inhibit the growth of some parasites include *Trypanosoma cruzi* and *Leishmania* species [166,183]. This antiparasitic effect may be linked to the activity of snake venom L-amino-acid oxidases [77,184]. For example, L-amino-acid oxidases from *Brothrops* snakes venoms provided a significant inhibition of parasitic growth of *Leishmania* species, *Plasmodium falciparum,* and *Trypanosoma cruzi* [185,186,187].

## 5. Snake Venoms for Drug Discovery

Development of new drugs represents one of the furthermost challenging activities of the pharmaceutical industry. Since the middle of the 20th century, a growing number of potential therapeutic agents have been extracted and isolated from plant, animal and microorganism toxins [7]. For example, snake venoms comprise a combination of biological active components that are involved not only in envenomation pathophysiology but also in the development of new drugs to treat many diseases [154]. While the preliminary effort with regard to snake venoms was to understand the effects of snakebites on humans and to elaborate the action of the toxins, snake venom components were also understood to be medical tools for thousands of years in Ayurveda, homeopathy and traditional/folk medicine for the treatment of a variety of pathophysiological conditions. In addition, snakes were considered the god of medicine in the ancient Greek world and the symbol of the snake is still used nowadays for medicine and pharmacy. In Ayurveda, cobra venom was used to treat joint pain, inflammation, and arthritis [188]. In addition, cobra venoms have been used for centuries by the Chinese to treat opium addiction and by the Indians who combined it with opium to treat pain. Moreover, other body fluids from snakes have been widely used in traditional Chinese medicine such as blood and bile duct [157]. With the advancement of modern biotechnology, the use of animal venoms components as a source of potential therapeutic values attracted the attention of pharmaceutical industry. In the past few decades, several potential drugs in use or in clinical trials have been isolated or derived from snake venom proteins (Table 2) [156].

### 5.1. FDA-Approved Drugs Derived from Snake Venom Proteins

In 1975, Captopril^®^ was the first successful and most reputed example of a drug developed on the basis of a snake venom component [189]. It was discovered by the Nobel Prize winner Sir John Vane and later commercialized by the pharmaceutical giant Squibb. The drug is a biomimetic of a bradykinin-potentiating peptide. It was isolated from the venom of the Brazilian arrowhead viper *Bothrops jararaca* and used to treat hypertension and cardiovascular disease by inhibiting angiotensin converting enzyme which is responsible for the conversion of angiotensin I to angiotensin II [190]. The drug was approved by the FDA in 1981 and used for the treatment of high blood pressure, renal disease in diabetics and heart failure after myocardial infarction [191]. Several generations of the drug have been developed by Squibb and other pharmaceutical companies [192].

Since the approval of captopril, snake venoms have become an important natural pharmacopeia of bioactive molecules that provide a good source of compounds for the development of new drugs. Aggrastat^®^ (Tirofiban) and Integrilin^®^ (Eptifibatide), two drugs based on snake venom disintegrins are available on the market as antiplatelet agents [193,194]. Aggrastat is a drug now marketed by Medicure Pharma in the US and Correvio International outside of the US but was originally developed by Merck. Aggrastat was designed to reduce the rate of thrombotic cardiovascular events such as a heart attack. It is an antiplatelet drug, which belongs to the platelet glycoprotein (GP) IIb/IIIa inhibitors [195]. It was developed based on the RGD sequence (Arg-Gly-Asp) motif from snake venom disintegrins isolated from the venom of *Echis carinatus* [196,197]. Disintegrins are a family of low molecular weight cysteine-rich proteins, originally purified from viperid venoms that usually contain the integrin-binding RGD motif [198]. The drug was approved by FDA on the 14 May 1998 and used for the treatment of heart attack patients.

Integrilin^®^ (Eptifibatide) was developed by Millennium Pharmaceuticals and co-promoted by Schering-Plough which are both now part of Merck and Takeda Pharmaceuticals. Integrilin is an injection given to patients with acute coronary syndrome to decrease the chance of a new heart attack or death, including patients undergoing percutaneous coronary intervention [199]. Integrilin is a peptide designed to mimic a small portion of the glycoprotein (GP) IIb/IIIa inhibitor barbourin found in the venom of the Southeastern pygmy rattlesnake (*Sistrurus miliarus barbouri*) [200]. The GP IIb/IIIa integrin plays a critical role in mediating platelet aggregation. However, Intergrilin unlike Aggrastat, is developed based on the KGD sequence (Lys-Gly-Asp) of the disintegrin from the Southeastern pigmy rattlesnake venom. The FDA approved Integrilin in 1998 and the drug is used for the treatment of patients with acute coronay syndrome [199].

Defibrase^®^/Reptilase^®^ (Batroxobin) is not approved clinically in the US, but has been approved for use in other countries. Batroxobin is a thrombin-like serine protease enzyme isolated from the snake venom of two subspecies *Bothrops atrox* and *Bothrops moojeni* [201]. Batroxobin strongly converts fibrinogen into fibrin through the release of fibrinopeptide A from fibrinogen. Outside of the US (largely in China), batroxobin is used to treat a range of disorders, including stroke, pulmonary embolism, deep vein thrombosis, myocardial infarction and perioperative bleeding. In the same way, Hemocoagulase^®^ is delivered from the venom of the Brazilian snake *Bothrops atrox*. It has been used in plastic surgery, abdominal surgery, and human vitrectomy [202]. Exanta^®^ (Ximelagatran) from cobra venom is a thrombin inhibitor anticoagulant that has been used as a blood thinner and thrombin inhibitor [203].

### 5.2. Toxin-Derived Drugs from Snake Venom Proteins in Clinical Trials and Stages of Development

Further, it is interesting to note that there are few toxin-based drugs that are presently being approved for phase III clinical trials and are at various stages of development with promising horizons of application in the US. Alfimeprase is a recombinant protein of the enzyme P-I metalloproteinase fibrolase with thrombolytic activity, originally isolated from the venom of southern copperhead snake (*Agkistrodon contortrix*). Alfimeprase was used for treatment of patients with acute peripheral arterial occlusion (PAO) [204]. Viprinex^TM^ (Ancrod) is a serine proteinase isolated from the venom of the Malayan pit viper (*Agkistrodon rhodostoma*). Viprinex is being tested as a defibrinogenating agent for use in the treatment of acute ischemic stroke to attenuate or block additional clot formation in this disease [205,206].

Toxins or toxin-designed drugs also have found their usefulness as diagnostic tools. Four Viperid snake venom enzymes are currently in use as diagnostic tools. For example, Protac^®^ is a serine proteinase isolated from *Agkistrodon contortix* venom, which activates plasma protein C. It is used in the determination of protein C and protein S levels in blood [207]. Botrocetin^®^ is a platelet aggregating protein from the venom of *Bothrops jararaca* that enhances the affinity of the von Willebrand factor A1 domain for the platelet receptor glycoprotein Ibalpha (GPIbalpha) [208]. The thrombin like serine proteinase RVV-V from *Vipera russelli* venom is an activator of factor V. Factor V is one of the components of the blood coagulation cascade. FVV-V is used to destabilize and selectively inactivate factor V in plasma [209]. Russell’s viper venom-factor X activator (RVV-X) is a P-IV metalloproteinase and has been identified as a main procoagulant enzyme involving coagulopathy, which might be responsible for changes in renal hemodynamics and renal functions [210]. Ecarin is a metalloprotease isolated from the venom of the saw-scaled viper (*Echis carinatus*) and used as prothrombin activator. Ecarin does not affect other clotting factors [211,212].

## 6. Conclusions

It may be concluded that only a small fraction of snake venom components have been identified, and continued technical improvements in the drug discovery field are likely to uncover many new therapeutic leads from snake venoms.

## Figures and Tables

**Table 1:** Antimicrobial activity of snake venoms.

Properties	Target Microbe	Factor (Protein/Peptide)	Source (Snake Specie)
**Antibacterial**	Gram-positive and gram-negative bacteria	L-amino acid oxidase	*Bothrop alternatus*, *Bothrop pirajai, Bothrop asper*, *Bothrop leucurus*, *Crotalus adamanteus, Daboia russelli russelli, Ophiophagus hannah* and *Pseudechis australis*
		Phospholipase (PLA_2_)	*Bothrop asper*, *Bothrop jararacussu*, *Bothrop pirajai*, *Bothrop moojeni*, *Bungarus faciatus* and *Crotalus durissus terrificus*
**Antiviral**	Measles, yellow fever and dengue viruses	Crotoxin and PLA_2_	*Crotalus durissus terrificus*
	Sendai virus	Cytotoxins	*Naja nigricollis*
	Human Immunodeficiency Virus (HIV)	L-amino acid oxidase	*Trimeresurus stejnegeri, Bungarus candidus*, *Naja naja* and *Naja kaouthia*
	*Hepatitis C virus* (HCV)	Crotoxin, PLA_2_ and crotapotin	*Crotalus durissus terrificus*
**Antifungal**	*Candida parapsilosis* and *Sporothrix schenckii*	Metalloproteinases and PLA2	*Crotalus durissus cumanensis*
		Crotamine	*Crotalus durissus terrificus*
**Antiparasitic**	*Leishmania* species, *Plasmodium falciparum* and *Trypanosoma cruzi*	L-amino-acid oxidases	*Brothrops* snakes

**Table 2:** Snake venom-based drugs in the market and in clinical trials.

Stage	Protein/Peptide	Lead Source	Pharmacology	Indication
**FDA Approved**	Captopril	*Bothrops jararaca*	Inhibiting angiotensin-converting enzyme.	Hypertension
	Aggrastat (Tirofiban)	*Echis carinatus*	Glycoprotein IIb/IIIa inhibitors.	Heart attack
	Integrilin (Eptifibatide)	*Sistrurus miliarus barbouri*	Glycoprotein (GP) IIb/IIIa inhibitors.	Acute coronary syndrome
	Defibrase/Reptilase (Batroxobin)	*Bothrops atrox* & *B. moojeni*	Converts fibrinogen into fibrin.	Stroke, pulmonary embolism, deep vein thrombosis and myocardial infarction
	Hemocoagulase	*Bothrops atrox*	Catalyzes the coagulation of the blood.	Plastic surgery, abdominal surgery, and human vitrectomy
	Exanta (Ximelagatran)	Cobra venom	Direct thrombin inhibitors.	Thromboembolic complications of atrial fibrillation
**In clinical trials**	Alfimeprase	*Agkistrodon contortrix*	Thrombolytic activity.	Acute peripheral arterial occlusion
	Viprinex (Ancrod)	*Agkistrodon rhodostoma*	Defibrinogenating agent.	Acute ischemic stroke
